# Supplementary material for: The Influence of Body Weight on Semen Parameters in Apis mellifera Drones
Source: Insects. 2022 Dec 11;13(12):1141. doi: 10.3390/insects13121141 (PMC9785928; doi:10.3390/insects13121141)
Supplement: Supplementary file 1 [file insects-13-01141-s001.zip › insects-1966227-supplementary.pdf]

Supplementary materials

Staining techniques used for honey bee drone spermatozoa

2.4.1. Eosin G 2% staining technique

A smear was made from the semen sample by spread of 5 µl semen on a degreased glass slide. After drying, Eosin G 2% was placed on the surface of the smear and kept covered with a film of this solution for two minutes. The stain was removed from the slide by successive immersions in tap water, after which the stained smear was air-dried.

2.4.2. Spermac staining technique

A drop of semen was spread on a dry and degreased glass slide, heated to 37°C and dried for 5 minutes at room temperature, after which the smear was fixed by immersing in the fixative solution for 5 minutes. At the end of the time, the slide was washed by immersion 6-7 times in distilled water, after which the excess water was removed by wiping the edges of the slide with absorbent paper. The slide was placed in stain A for 1-2 minutes and washed, after which the slide was placed in stain B for 1 minute after which it was washed again. Then the slide was inserted into stain C for 1 minute and, after washing, it was dried at room temperature.

2.4.3. Diff-Quik staining technique

The technique recommended by the World Health Organization in the WHO laboratory manual 2010 [43], slightly modified, was used. The smear from the sperm sample was dried at room temperature after which it was immersed in the fixative solution of the kit for 15 seconds. This was followed by the immersion in solution I, for 10 seconds, and immersion in solution II, for 10 seconds, after which washing was carried out in tap water, performing 10-15 plunges. After each solution, the slide was placed upright on absorbent paper to remove excess stain.
